# Split Voice, Whole Self: The Philosophical Significance of Internal Dialogue in Psychotherapy

**DOI:** 10.1192/j.eurpsy.2026.11898

**Published:** 2026-07-31

**Authors:** M. Corrielus, M. O’Neill, A. Eason, B. Carr

**Affiliations:** 1 College of Medicine; 2 Department of Psychiatry; 3 Department of Anesthesiology, University of Florida, Gainesville, United States

## Abstract

**Introduction:**

Internal dialogue, particularly second person self-address (e.g “Why are you doing this?”) has long been noted in clinical psychotherapy where it manifests as both a symptom (fragmented voices in splitting or dissociation) and a tool of integration. Despite its relevance to therapeutic change, the philosophical structure of internal dialogue remains underexplored in psychiatry. Thinkers such as This project investigates the role of internal dialogue and attempts to offer a unified account linking phenomenology, therapeutic processes, neurological mechanisms and philosophical concepts of selfhood.

**Objectives:**

To synthesize evidence on neurobiological bases of inner speech, (1) neurobiological bases of inner speech, (2) its dysfunction in split defenses and affective fragmentation, (3) its role as a mechanism of therapeutic change, and (4) its philosophical grounding in dialogic selfhood (Jaspers, Bakhtin, Vygotsky, Benjamin).

**Methods:**

A selective literature review and conceptual synthesis were conducted, integrating philosophical texts, psychotherapy process studies, neuroimaging research on self-referential processing, and anonymized clinical vignettes observed during medical school clerkships, with attention to second-person self-address as a transitional marker.

**Results:**

Dialogic inner speech recruits language regions alongside default mode and self-referential networks. Disruptions in these circuits contribute to phenomena such as misattributed voices in psychosis and rigid, punitive inner critics in depression. In borderline and dissociative states, split-off self-voices remain unintegrated, sustaining affective instability. Psychotherapy process research shows that the emergence of second-person self-address (eg “You need to calm down”) often marks a turning point, enabling patients to observe themselves and create an internal conversation between parts that were previously disconnected. Clinical techniques such as chair dialogues, parts work, and guided imagery capitalize on this dynamic by fostering constructive self-to-self communication.

**Conclusions:**

This analysis proposes that internal dialogue should be recognized not only as a coping strategy, but as a philosophically rich marker of recovery. It challenges models that treat intrapsychic conflict as pathology and invites renewed attention to voice, address, and dialogic structure in psychotherapy. Further research may explore how therapeutic interventions can facilitate this inner dialogic emergence.

**Disclosure of Interest:**

None Declared

